# Protocol for a proof-of-concept observational study evaluating the potential utility and acceptability of a telemedicine solution for the post-anesthesia care unit

**DOI:** 10.12688/f1000research.26794.1

**Published:** 2020-10-20

**Authors:** Thaddeus P. Budelier, Christopher Ryan King, Shreya Goswami, Anchal Bansal, Stephen H. Gregory, Troy S. Wildes, Joanna Abraham, Sherry L. McKinnon, Amy Cooper, Ivan Kangrga, Jackie L. Martin, Jr., Melissa Milbrandt, Alex S. Evers, Michael S. Avidan

**Affiliations:** 1 Department of Anesthesiology, Washington University School of Medicine, St. Louis, MO, 63110, USA; 2 Institute for Informatics, Washington University School of Medicine, St. Louis, MO, 63110, USA; 3 Department of Perioperative Services, Barnes-Jewish Hospital, St. Louis, MO, 63110, USA; 4 Department of Internal Medicine, Washington University School of Medicine, St. Louis, MO, 63110, USA; 5 Department of Developmental Biology, Washington University School of Medicine, St. Louis, MO, 63110, USA

**Keywords:** Telemedicine, Post-Anesthesia Care Unit, Protocol, Proof-of-Concept, Observational Study

## Abstract

**Introduction: **The post-anesthesia care unit (PACU) is a clinical area designated for patients recovering from invasive procedures. There are typically several geographically dispersed PACUs within hospitals. Patients in the PACU can be unstable and at risk for complications. However, clinician coverage and patient monitoring in PACUs is not well regulated and might be sub-optimal. We hypothesize that a telemedicine center for the PACU can improve key PACU functions.

**Objectives: **The objective of this study is to demonstrate the potential utility and acceptability of a telemedicine center to complement the key functions of the PACU. These include participation in hand-off activities to and from the PACU, detection of physiological derangements, identification of symptoms requiring treatment, recognition of situations requiring emergency medical intervention, and determination of patient readiness for PACU discharge.

**Methods and analysis: **This will be a single center prospective before-and-after proof-of-concept study. Adults (18 years and older) undergoing elective surgery and recovering in two selected PACU bays will be enrolled. During the initial three-month observation phase, clinicians in the telemedicine center will not communicate with clinicians in the PACU, unless there is a specific patient safety concern. During the subsequent three-month interaction phase, clinicians in the telemedicine center will provide structured decision support to PACU clinicians. The primary outcome will be time to PACU discharge readiness determination in the two study phases. The attitudes of key stakeholders towards the telemedicine center will be assessed. Other outcomes will include detection of physiological derangements, complications, adverse symptoms requiring treatments, and emergencies requiring medical intervention.

**Registration: **This trial is registered on clinicaltrials.gov, NCT04020887 (16 ^th^ July 2019).

## Introduction

After invasive procedures in the operating room (OR) or other procedure rooms, patients are usually transferred to a post-anesthesia care unit (PACU) for high acuity monitoring. The PACU period is important for patients, especially since they often are still in a vulnerable state ^1, 2^. Patients are prone to peri-procedural and post-anesthetic complications including dehydration, anemia, coagulopathy, bleeding, hypothermia, delirium, respiratory depression, airway obstruction, bronchospasm, hypotension, kidney injury, arrhythmias, metabolic acidosis, hypoxemia, glucose and electrolyte abnormalities, atelectasis, and pulmonary edema ^3, 4^. These complications must be recognized and appropriately managed by PACU clinicians. Furthermore, PACU clinicians need to identify and manage patients’ adverse symptoms including pain, nausea, urine retention, weakness, and itching, which are common after invasive procedures, whether with or without general anesthesia.

The ideal PACU environment provides close monitoring and prompt rescue for peri-procedural complications, while also efficiently transferring patients to their next phase of care. For example, when patients deteriorate in the PACU, it is important to recognize this early, intervene appropriately, and arrange transfer to a higher acuity area, such as an intensive care unit, when warranted.

PACU clinicians are responsible for several clinical and organizational tasks ^5^ including patient monitoring and treatment, promoting patient throughput, conducting hand-offs to and from the PACU, and documenting patient care information during the recovery period. As a result, PACU nurses and doctors can feel overwhelmed, and may not always be able to treat symptoms adequately, diagnose physiological derangements accurately, and detect patient deterioration expeditiously. Furthermore, in this high-pressure, high-turnover environment, communication among clinicians is often compromised, resulting in unreliable care coordination. Patient satisfaction with PACU care varies, as the recognition and prompt treatment of symptoms depends on the availability of assigned clinicians.

The necessity of operating room throughput creates a constant pressure on PACU clinicians to discharge patients rapidly, sometimes before they have recovered sufficiently. This workflow pressure can potentially compromise quality of care and patient safety. Nurses provide the majority of PACU care, typically for no more than two patients at a time during the initial phase of PACU care, in accordance with the American Society of PeriAnesthesia Nurses (ASPAN) guidelines ^6^. Furthermore, physicians with competing responsibilities often provide oversight in the PACU. For example, a physician who has responsibility for patient assessment and management in the PACU is often simultaneously overseeing anesthetic care in operating rooms or other procedural suites. Surgical clinicians also participate in aspects of PACU care, but are often simultaneously engaged in surgical care of other patients. In addition, the coverage and oversight models can vary considerably across different PACUs, and even within the same PACU over the course of a single day. This is in stark contrast to other high acuity patient care settings, such as operating rooms and intensive care units, where roles and responsibilities of various clinicians are well defined, and staffing models are established.

In this protocol, we describe a proof-of-concept study in perioperative telemedicine that aims to demonstrate the (i) potential utility and (ii) acceptability of integrating telemedicine into the PACU environment. This proof-of-concept study will be conducted in the PACU located in Parkview Tower in Barnes-Jewish Hospital (BJH). If this proof-of-concept proves to be successful, we intend subsequently to show the impact of such a telemedicine solution on safety, quality of care, efficiency, and ultimately postoperative outcomes. Our specific aims for the proposed proof-of-concept study are:

### Aim 1 – Demonstrate the potential utility of a telemedicine center for the PACU, to assist with PACU functions

*We hypothesize that clinicians in the telemedicine center for the PACU will:*

1a. Detect physiological derangements and complications
1b. Identify adverse symptoms requiring treatment
1c. Recognize situations requiring emergency medical intervention
1d. Determine when patients are ready for PACU discharge
1e. Participate meaningfully in hand-off activity from the OR to the PACU

### Aim 2 – Identify barriers to and facilitators for the implementation of a telemedicine center for the PACU, as perceived by key stakeholders

We will assess attitudes of key stakeholders towards a telemedicine center for PACU. The key stakeholders will include PACU nurses, anesthesiologists, surgeons, hospital administrators, and PACU-telemedicine center clinicians.

## Methods

### Ethical statement

This proof-of-concept study has been approved and granted a waiver of informed consent for all patients and a waiver of written consent for participants enrolled by the Human Research Protection Office at Washington University in St. Louis (HRPO#201901180) and is registered at clinicaltrials.gov ( NCT04020887, 16 ^th^ July 2019). It is infeasible to conduct this proof-of-concept study without a waiver of consent. Additionally, this study has been determined to involve no more than minimal risk to participants, as study participation would not deviate from or delay current standards of peri-anesthesia care.

### Study setting, design, and participants

The study will be conducted at Barnes-Jewish Hospital (BJH) in St. Louis, Missouri, a large tertiary care academic medical center.

We will conduct a single center prospective before-and-after proof-of-concept study to evaluate a telemedicine center for the PACU. Adults (18 years and older) undergoing elective surgery at Barnes Jewish Hospital in St. Louis, Missouri will be enrolled. Approximately 500 patients will be enrolled in this study over a six-month duration, with an estimated 250 patients allocated to each phase of the trial. The first phase is an Observation phase and the next phase is an Interaction phase. More information on these phases is provided below.

Both the “Good ReseArch for Comparative Effectiveness” (GRACE) checklist ^7^ and PICOTS framework ^8^ ( Table 1) were used in designing this study. The conduct and reporting of this observational study will follow the “Reporting of studies Conducted using Observational Routinely-collected health Data” (RECORD) ^9^ statement and the “Strengthening the Reporting of Observational Studies in Epidemiology” (STROBE) ^10^ statement guidelines for reporting observational studies.

**Table 1.** PICOTS Framework.

**PICOTS typology for a comparative effectiveness research protocol**	
Population	Adult (18 years and older ) patients undergoing elective surgery
Intervention	Telemedicine center for PACU
Comparator	Current post-anesthesia care unit practice
Outcomes	(i) potential utility, and (ii) acceptability of integrating telemedicine in the post-anesthesia care unit environment
Timing	6-month study duration
Setting	Hospital environment – Barnes-Jewish Hospital, St. Louis, Missouri

PACU, post-anesthesia care unit.

### Primary intervention: telemedicine center for PACU

**Figure 1.**
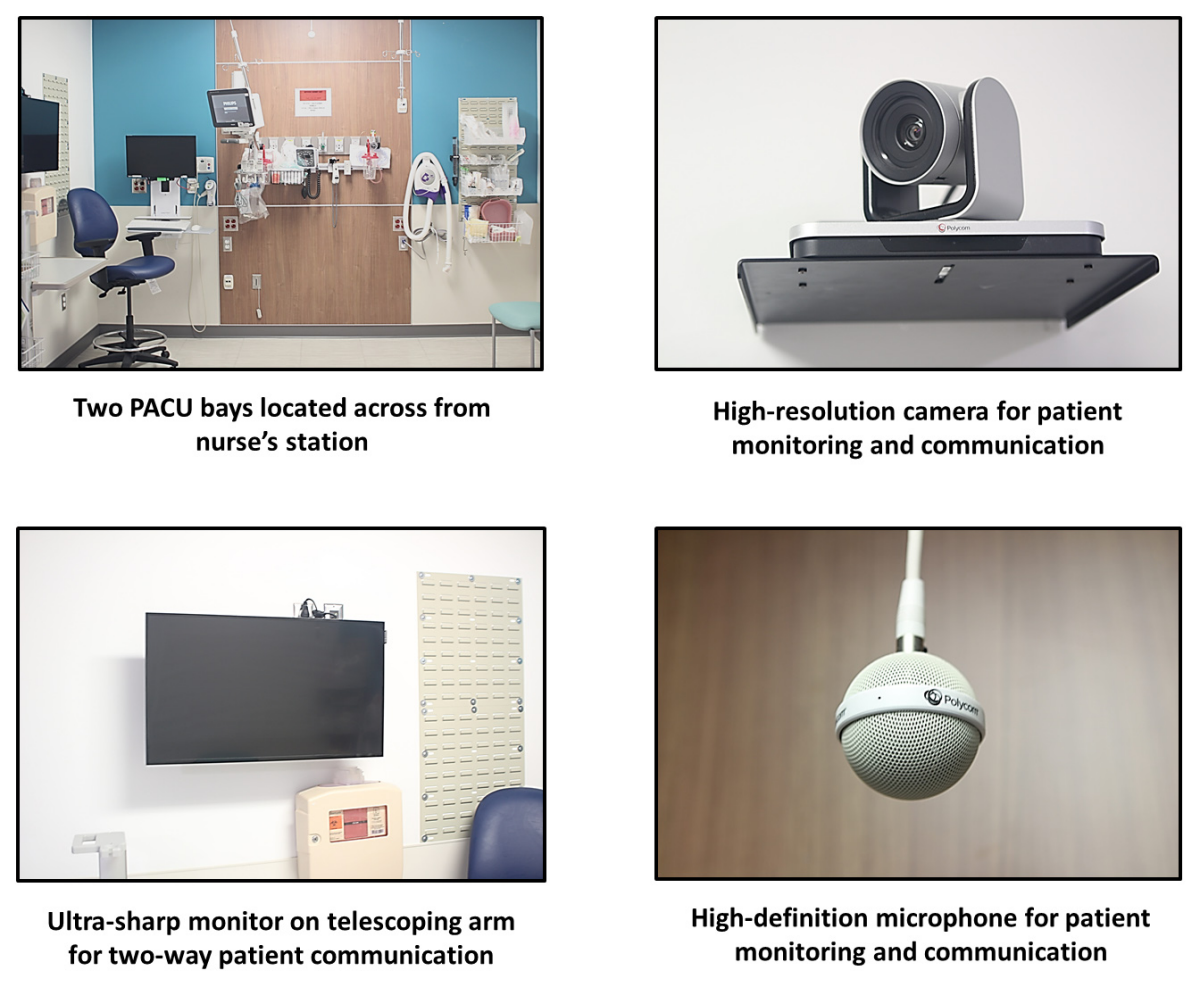
Image of post-anesthesia care unit bay in Barnes-Jewish Hospital with two-way video communication.

Two bays in Barnes Jewish Hospital (BJH) in St. Louis, Missouri, will be equipped for telemedicine interaction ( Figure 1). Video cameras and monitors have been installed in each of these bays to allow for remote monitoring, as well as two-way video communication during the interaction phase. The telemedicine center is staffed by attending anesthesiologists along with certified registered nurse anesthetists (CRNAs), anesthesiology residents, and student registered nurse anesthetists (SRNAs), and is currently providing evidence-based support to clinicians in the operating rooms ^11– 14^.

**Figure 2.**
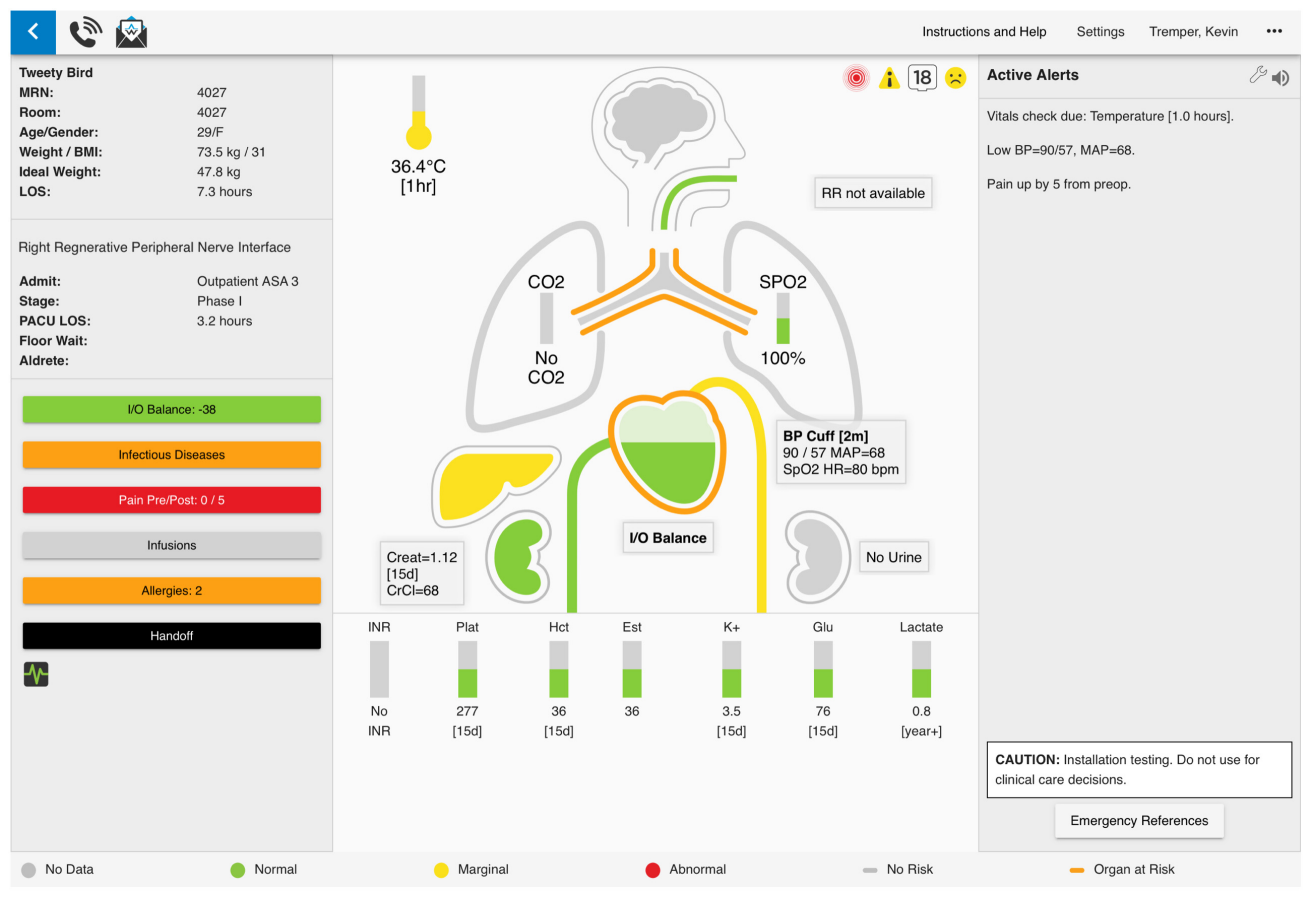
AlertWatch® decision-support software, customized for the post-anesthesia care unit (PACU) environment.

A station in our telemedicine center will be designated for monitoring patients assigned to the two PACU bays during this proof-of-concept study. Patient information flows to the telemedicine center through the electronic health record (EHR), physiological waveform tracings, and direct video observation. A version of AlertWatch® (AlertWatch, Ann Arbor, Michigan) decision-support software, customized for the PACU environment ( Figure 2), will assist clinicians in the telemedicine center in performing core PACU-related functions remotely ( *see Aim 1*).

### Aim 1 – Demonstrate the potential utility of a telemedicine center for the PACU, to assist with PACU functions

*The assessments in relation to PACU functions will include:*

1a. Detection of physiological derangements in PACU patients
1b. Identification of symptoms requiring treatment in PACU patients
1c. Recognition of situations requiring emergency medical intervention
1d. Determination of patient readiness for PACU discharge
1e. Participate meaningfully in hand-off activities

***Observation phase (three months).*** In the first three months (the Observation phase) of this proof-of-concept study, a telemedicine center for the PACU will monitor patients assigned to two PACU bays. Both the telemedicine center and nurses caring for patients in the PACU bays will separately document physiological derangements ( Table 2), treatable symptoms ( Table 3), or a situation requiring urgent medical intervention (telemedicine center only; Table 4) during the PACU stay. Clinicians in the telemedicine center will assess when the patient meets discharge criteria, based on the modified Aldrete scale ^15^ and their clinical judgment. They will document the time that discharge criteria are met, the modified Aldrete scale score at this time, and any additional relevant information. If clinicians in the telemedicine center judge that they are unable to determine a patient’s readiness for discharge, they will document their reasons ( Table 5). Clinical judgment will be used in determining appropriate discharge parameters for patients with pre-existing conditions. The telemedicine center clinicians will document each patient’s information outlined in Table 2– Table 5 directly into REDCap ^TM^ (a secure web application for managing online surveys and databases) and AlertWatch. After a patient has been discharged from the PACU, the PACU nurse will fill out a form providing information outlined in Table 2– Table 5. This includes information on physiological derangements, treatable symptoms, and discharge information. This form will be collected by the research team, and the information in the form will be documented in REDCap. During this phase of the study, clinicians in the telemedicine center will not communicate with clinicians in the PACU (nurses or physicians), unless there is a patient safety event.

**Table 2.** Physiological derangements and complications.

Did the patient have any of the following physiological derangements:	Definition: (for study purposes)
Persistent confusion / delirium	
Tachycardia	HR >120/min
Bradycardia	HR <45/min
New onset atrial fibrillation	
Respiratory depression	<8 respirations per minute
Hypoxemia	<90% 02 Saturation
Hypotension	MAP <55
Weakness	<5/5 power in limbs
Emesis / vomiting	
Hyperglycemia	Glucose >200mg/dL
Hypothermia	Temperature < 35.5°C
Low urine output (for PACU stay >4h)	<0.5 ml/kg per hour

HR, heart rate; MAP, mean arterial pressure; PACU, post-anesthesia care unit.

**Table 3.** Symptoms requiring treatment.

**Did the patient complain of the following symptoms:**	
Dizziness or lightheadedness	Difficulty breathing
Nausea	Shivering
Severe pain (Numerical Rating Scale >7/10)	Itching
Chest pain unrelated to surgery	

**Table 4.** Emergency medical interventions.

**Did the telemedicine center contact PACU clinicians for any of the following** **interventions:**	
Intubation	Unplanned transfusion
Assisted ventilation	Naloxone administration
Cardiopulmonary resuscitation	Return to OR
Cardioversion	Other (free text box)
**If Yes, please check all that apply:** □ PACU nurse already aware of the situation □ PACU nurse unaware of the situation □ PACU nurse disagreed with the assessment □ PACU nurse had already spoken to the supervising physician regarding the situation □ Other (please describe):	

*Only the telemedicine center will document the detection of urgent situations. OR, operating room; PACU, post-anesthesia care unit.

**Table 5.** Patient discharge readiness.

At what time did the patient sufficiently recover to be discharged (case attending anesthesiologist contacted for discharge)?	
PACU Nurses	Telemedicine Center for PACU
Time anesthesiologist is contacted for discharge evaluation	Time ready for discharge
Aldrete Score at discharge	Aldrete Score at discharge
	Unable to determine patient’s readiness for discharge
**Modified Aldrete Scale Component and Scoring** **Parameters**	**If Unable to fully assess, select** **reason(s) why (Checkbox)**
**Respiration**	□ More patient information needed
2 – Able to take deep breath and cough	□ Equipment issues
1 – Dyspnea / Shallow Breathing	□ Patient cooperation
0 – Apnea	□ Other (free text box)
**0 _2_ Saturation**	
2 – Maintains > 92% on room air	
1 – Needs 0 _2_ inhalation to maintain 0 _2_ saturation > 90%	
0 – Saturation <90% even with supplemental 0 _2_	
**Consciousness**	
2 – Fully awake	
1 – Arousable on calling	
0 – Not responding	
**Circulation**	
2 – BP ± 20mmHg pre-op	
1 – BP ± 20-50mmHg pre-op	
0 – BP ± greater than 50mmHg pre-op	
**Motor activity**	
2 – Able to move 4 extremities voluntarily or on command	
1 – Able to move 2 extremities voluntarily or on command	
0 – Able to move 0 extremities voluntarily or on command	

BP, blood pressure; PACU, post-anesthesia care unit.

***Interaction phase (three months).*** In the three months following the observation phase, clinicians in the telemedicine center will interact with patients and clinicians associated with the designated PACU bays using audio-visual technology. PACU clinicians and clinicians in the telemedicine center will become a “fused” team, and the telemedicine center will continue to document information on physiological derangements ( Table 2), treatable symptoms ( Table 3), situations requiring urgent medical intervention ( Table 4), and discharge readiness ( Table 5).

The telemedicine center clinicians will assess patients’ discharge readiness throughout their PACU stay. A modified Aldrete scale along with clinical judgment will guide the telemedicine center clinicians in determining readiness for discharge ( Table 5). After discharge readiness has been determined by the telemedicine center, the attending anesthesiologist in the telemedicine center will document discharge readiness in AlertWatch and REDCap, and contact the relevant anesthesiologist. The telemedicine center for PACU will document when this information was communicated. At any point clinicians in the telemedicine center might decide to contact PACU clinicians (nurse or physician) if they have specific concerns regarding patients. If the telemedicine center clinicians feel that they cannot adequately assess a patient’s clinical status, they will notify the PACU clinicians. This will be documented together with a relevant explanation ( Table 5).

Final determination and sign-off regarding discharge suitability will be made by the anesthesiologist in the PACU. With this proof-of-concept research project, there will be no change in relation to which clinicians have responsibility for decision making and clinical care. The telemedicine center clinicians will not write any orders in the medical record, and will provide opinions only to physicians and nurses who are responsible for patient care in the PACU. The responsibility to call for help when patients are deteriorating will remain with the PACU nurses, as is the current standard in that environment. The notion is that the telemedicine center will not lead to any decrement in the care that PACU patients are currently receiving from nurses and physicians in that environment.

**Figure 3.**
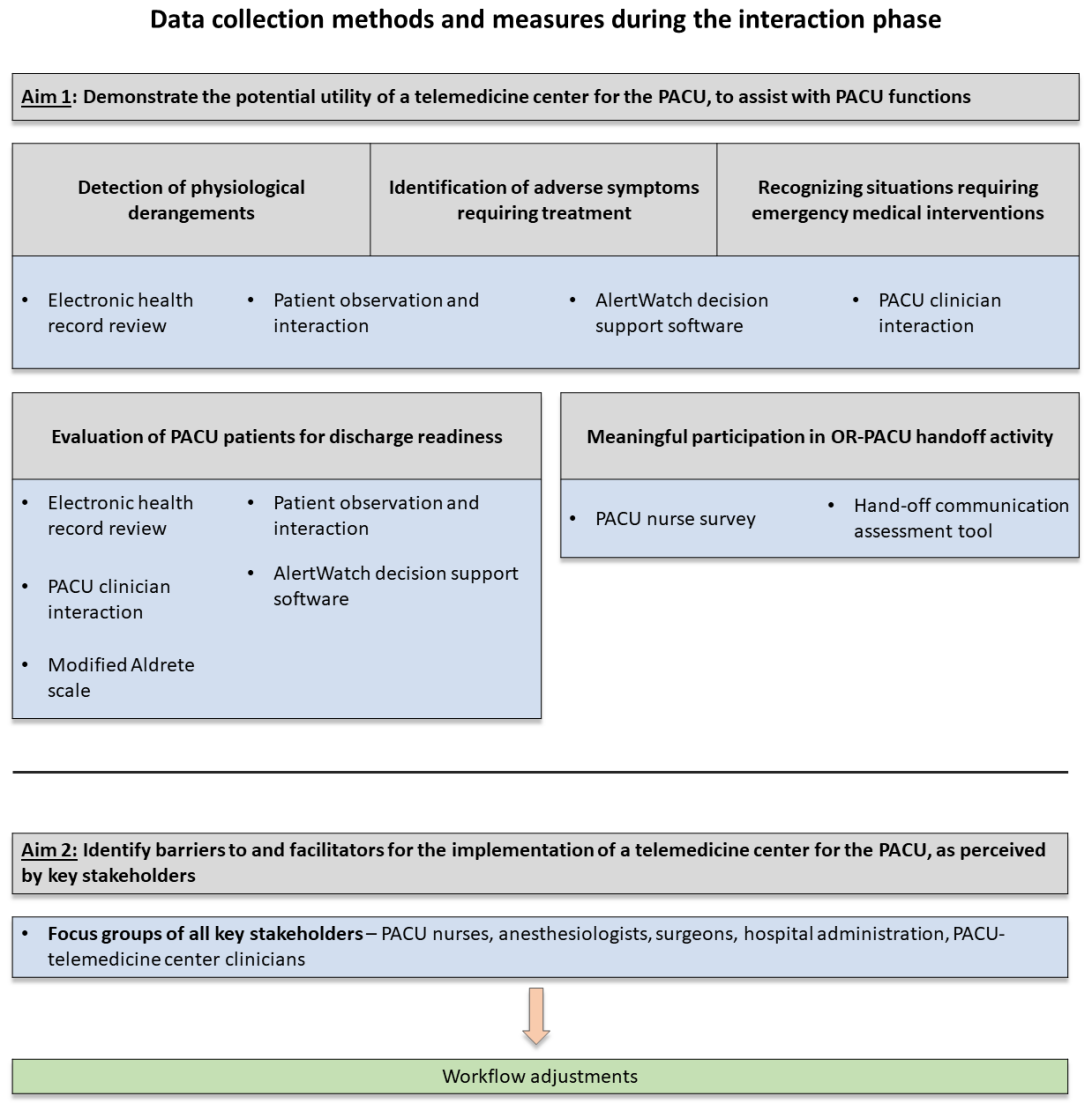
Overview of data collection methods and outcome measures during the interaction phase of a before-and-after proof-of-concept study for a telemedicine center for the post-anesthesia care unit (PACU).

The successful integration of the telemedicine center into each of the core PACU functions will be measured in the following ways:

*Physiological derangements* – Success will be measured (in the observation phase) by the ability of the telemedicine center clinicians to identify physiological derangements as they are occurring in the PACU. The extent to which the telemedicine center clinicians can identify these physiological derangements will be measured by comparing PACU nurse and telemedicine center assessment surveys ^16^ for each patient ( Figure 3). 

*Symptom identification and management* – Success will be measured (in the observation phase) by the ability of the telemedicine center clinicians to identify treatable symptoms as they arise in the PACU. The extent to which the telemedicine center clinicians can identify these treatable symptoms will be measured by comparing PACU nurse and telemedicine center assessment surveys ^16^ for each patient ( Figure 3). 

*Emergency situations* – Success will be measured (in the observation phase) by the ability of the telemedicine center clinicians to identify situations requiring emergency medical intervention as they are occurring in the PACU. By construction, any time the telemedicine center feels that an emergency situation is present, preserving patient safety mandates contacting the bedside clinician. During each such contact, the telemedicine center clinician will ask if the PACU nurse was already aware of the situation, disagreed with the assessment, and had already spoken to the supervising physician regarding it. The occurrence of emergency medical situations will be extracted from the electronic health record, and the agreement between telemedicine center and PACU nurse assessments will be quantitated.

*PACU discharge* – Success will be measured by the ability of the telemedicine center clinicians to identify when patients are ready for discharge (observation phase [without communication] and interaction phase [active communication with patient and PACU clinicians]) ( Figure 3). The impact of the telemedicine center on this key function will be examined based on feedback from key stakeholder focus groups (interaction phase; *see Aim 2*). The difference between sign-out times in the observation and the interaction phases will be compared.

***Hand-off activity.*** The telemedicine center clinicians will participate in hand-off activities to and from the PACU. This includes ensuring appropriate transfer of information from operating rooms to the PACU. The telemedicine center clinicians will remotely join the hand-off conversations, and review patients’ medical history and intraoperative course to identify potential missed transfer of information.

During the observation phase, the telemedicine center clinicians will observe the hand-off workflow, gain familiarity with the current hand-off routine, and identify possible areas of missed information transfer where the telemedicine center clinicians may have adjunct utility. An example of potential adjunct utility would be communicating the importance of appropriate insulin and glucose management in the PACU for a patient with type I diabetes.

In the interaction phase of the study, the telemedicine center clinicians will try to fill gaps in information transfer during the hand-off procedure. In addition to remotely joining the hand-off conversation, the telemedicine center clinicians will share pertinent additional patient or procedural information, especially if this could inform the patient’s PACU medical treatment. After the completion of the hand-off procedure, the PACU nurse who interacted with the telemedicine center clinicians will complete a short survey ^16^ to assess the telemedicine center’s involvement in that patient’s transfer of care.

The successful integration of the telemedicine center clinicians’ hand-off activity will be measured in the following way:

*Hand-off activity* – Success will be measured (in the interaction phase) by the ability of the telemedicine center clinicians to join and contribute meaningfully to the hand-off discussion. The impact of the telemedicine center clinicians on this key function will be determined from feedback from key stakeholder focus groups ( *see Aim 2*) and PACU nurse surveys ^16^. These stakeholders will comment on utility of the telemedicine center’s involvement and provide suggestions for improvement. A binary assessment of hand-off adequacy will be provided by the PACU nurse hand-off survey ^16^. The telemedicine center clinician will use a hand-off content checklist ^16^ to record the number of mandatory items not discussed and number of recommended non-mandatory items discussed. For each of the observation and intervention phases, for 50 randomly selected cases a trained observer (not the participant in hand-off) will use the hand-off communication assessment tool ^16^ of Weinger and others ^17^ substituting the telemedicine center hand-off content checklist. A run-in phase of one month during the intervention will elapse before any of the 50 detailed communication evaluations are performed.

### Aim 2 – Identify barriers to and facilitators for the implementation of a telemedicine center for the PACU, as perceived by key stakeholders

We will assess the attitudes of key stakeholders in order to identify barriers to and facilitators for implementation of a telemedicine center for the PACU. ( Figure 3)

***Stakeholder focus groups.*** We will conduct focus groups with stakeholders to gain insights regarding their perceptions of barriers and facilitators related to the above-noted PACU functions before and after the implementation and use of a telemedicine center for the PACU. We will also gather perspectives from the stakeholders on the role and impact of the telemedicine center on their individual and team workflows in the PACU and between units during care transitions. Focus group participants will include nurses, anesthesiologists, surgeons, hospital administrators, and PACU telemedicine center clinicians. Our focus groups will be homogeneous in order to understand the clinician workflow based on their professional role, and their use of the telemedicine center in supporting their role and responsibilities. Each focus group will comprise five to six participants. This will allow in-depth discussions of the workflow problems and unintended consequences caused by the implementation and use of the telemedicine center for the PACU. The focus group sessions will be guided by a semi-structured interview guide focused on the following themes: (1) PACU core functions, (2) PACU patient workflow, (3) PACU clinician activities and tasks, (4) tools and technologies used to support the PACU workflow, (5) major barriers to PACU functions, (6) use of a telemedicine intervention as a potential mechanism to support effective and efficient functioning of the PACU. We plan to conduct 6-8 focus group sessions (four pre-intervention during observation phase, and four post-intervention during interaction phase) or until data saturation is attained.

### Study size

**Figure 4.**
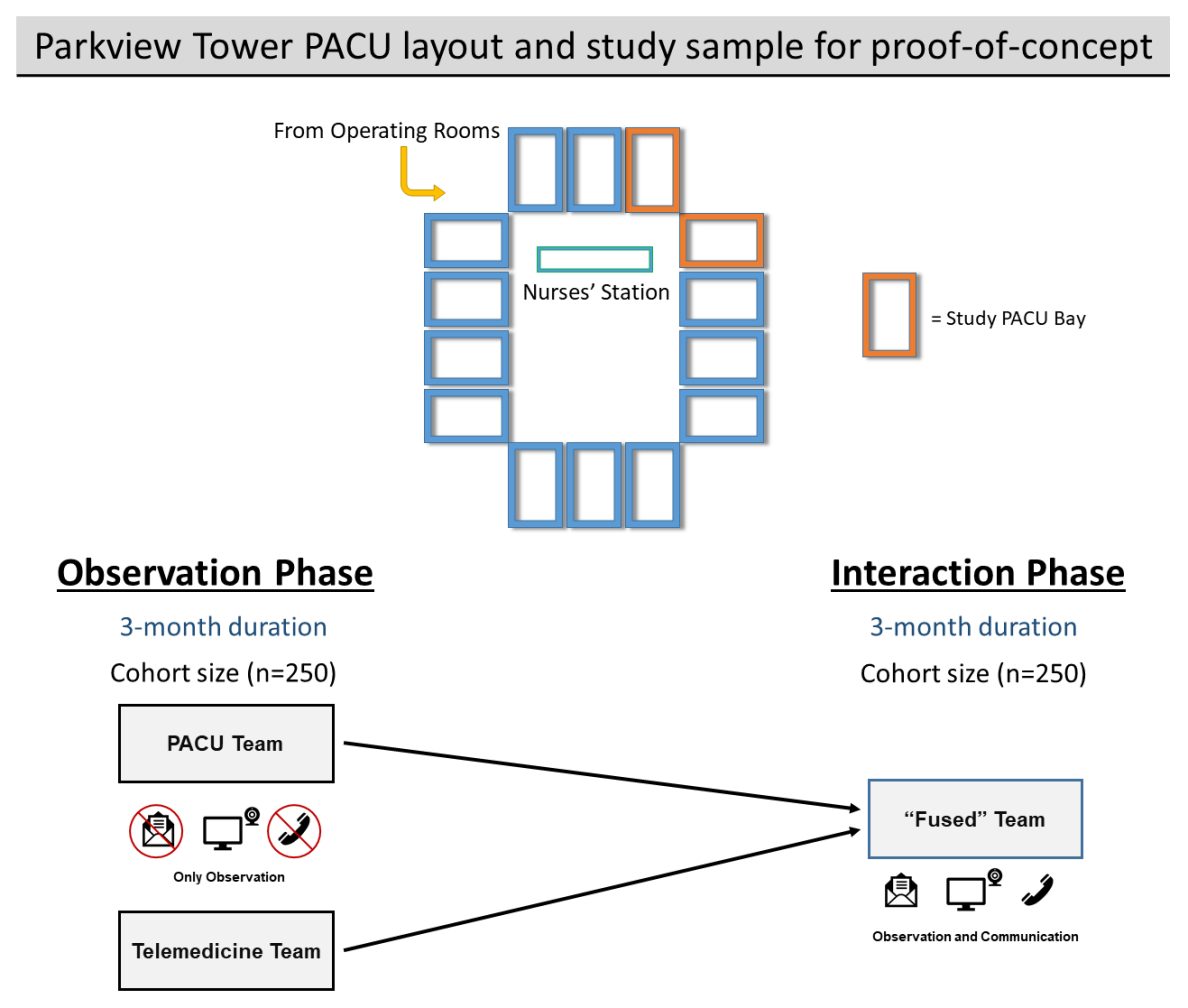
Allocation of sample size and post-anesthesia care unit (PACU) layout for proof-of-concept study.

Patients are allocated to PACU bays according to the discretion of the nurse in charge of the PACU. Currently, approximately two patients per day are cared for in each bay in the participating PACU. Therefore, the telemedicine team will monitor approximately four patients per day over the course of the proof-of-concept study. We estimate that 500 patients will be included in this proof-of-concept study (250 per monitored phase) ( Figure 4).

### Statistical methods

***Primary outcome.*** This is a proof-of-concept study and will only address surrogate outcomes. The primary outcome (time to PACU discharge readiness) will use two comparison groups. First, historical controls will be drawn from the observation phase. A propensity score for inclusion into the study will be generated as a function of (minimally) surgery performed, day of week, time of day, age, and sex. 3:1 matched control patients will be included. The outcome will be analyzed with interrupted time series methods with flexible functions of calendar time used to adjust for secular trends; the study hypothesis is a non-zero discontinuity at telemedicine implementation. That is, if Y _i_ is the outcome for the ith patient at time t _i _with covariate vector X _i_, while the implementation time is t _0_, and I() is the indicator function,

$$Y_{i}=f_{1}\left(t_{i}\right)I\left(t_{i}<t_{0}\right)+f_{2}\left(t_{i}\right)I\left(t_{i}\geq t_{0}\right)+X_{i}\beta +\in H_{0}:f_{1}\left(t_{0}\right)=f_{2}\left(t_{0}\right)$$

where f _1_ and f _2_ are smooth functions. Other patient factors known to strongly influence PACU length of stay (age, ASA physical status, number of co-morbidities, morbid obesity, obstructive sleep apnea, surgical specialty, primary anesthesia type, history of postoperative nausea and vomiting, preoperative pain, and scheduled case duration) will be included as covariates. The minimization criteria will be least squares or trimmed least squares or other robust criteria if there are substantial outliers. Outcomes will be examined for residual auto-correlation, and if non-negligible, auto-correlation robust standard errors (such as Newey-West errors) and an ARIMA model will be reported. Confidence intervals will be generated by non-parametric bootstrap sampling where possible. No adjustment will be made for matching, but bootstrap methods will respect the matched “units.” P-values will be generated both by likelihood ratio tests and by using non-deployment times as a null distribution; that is, we will run the same analysis looking for discontinuity at times remote from the true implementation time. We will conduct sensitivity analyses with transformations of the outcome variable. We will use an excluded run-in period of one month as a sensitivity analysis. Because hospital length of stay is unlikely to be meaningfully affected by a telemedicine center for the PACU, but does track overall acuity and surgical severity, we will use hospital length of stay as a control time series.

Contemporaneous control patients will also be gathered. A propensity score for study inclusion will be generated as a function of (minimally) surgery performed, calendar time, time of day, age, and sex. 3:1 matched control patients will be included. Differences will be analyzed by t-tests using permutation calibration. Confidence intervals on the difference in mean time to discharge readiness will be generated by nonparametric bootstrap. We will include a sensitivity analysis where the interrupted time series method includes historical and contemporaneous control patients with the treatment indicator T for study patients,

$$Y_{i}=f_{1}\left(t_{i}\right)I\left(t_{i}<t_{0}\right)+f_{2}\left(t_{i}\right)I\left(t_{i}\geq t_{0}\right)+X_{i}\beta +T_{i}+\in .$$

Based on data from our EHR, patients are currently in PACU for a mean of 150 min (standard deviation = 65 min) before they are determined to be suitable for discharge. Based on these values, with 250 patients in each phase (observation and interaction), this observational before and after study will have >70% power with an alpha <0.005 and > 90% power with an alpha <0.05 to detect a mean decrease in 20 min (from 150 min to 130 min) to PACU discharge readiness time. Statistical testing will be with appropriate statistical software. Using non-parametric bootstrap of historical data and a 3:1 control sampling ratio, the average standard error on the difference in means under the null hypothesis was 5.5 minutes, giving an anticipated 95% confidence interval width of 22 minutes. A somewhat larger standard error will be encountered when adjusting for covariates or secular trends; however, this suggests that we will be able to resolve differences in PACU readiness times of 20–25 minutes. This difference of approximately a third a standard deviation is usually regarded as a “small-moderate” sized effect.

***Secondary outcomes.*** Hand-off quality assessment from the PACU nurse binary survey response will be analyzed using a logistic regression model adjusting for surgical service, age, and sex. Because observation resources are required for hand-off evaluations, no matching will be performed, and no contemporaneous controls will be gathered. Adjusted differences in rates of inadequate hand-off will be summarized with 95% confidence intervals and model-based p-values. Observed reported hand-off communication quality will be presented as a purely descriptive result.

The accuracy of physiologic, symptom, and status assessments is less straightforward to analyze. At the heart of the proposal is the belief that telemedicine assistance will detect some abnormalities not caught (or caught later) by the bedside team and detect that the patient has adequate status for PACU discharge before the bedside nurse. Using the bedside assessment as a gold standard is therefore limited. Similarly, although we believe that abnormalities detected by either bedside or telemedicine are unlikely to be false positives, we have no way of assuring that. We also cannot reliably determine the timing of the bedside nurse’s detection of an abnormality, as they may document it much later if they believe it does not require an immediate intervention.

Each status assessment event can occur multiple times for each patient; however, we are unlikely to accurately capture the bedside nurse’s impression of the number of times an event occurred. We will therefore binarize the presence of each assessment type and display confusion matrices (count tabulations) for each assessment type, which we will summarize with Jaccard indicies. The “null hypothesis” that these measures do not agree at all is not meaningful or the subject of this study. As described above, neither is a directional superiority hypothesis possible to evaluate. Final Aldrete scores will be assessed with pearson correlation, and a t-test of the difference in scores presented. Differences in ready-for-discharge times will be summarized as mean and standard deviation, with the null hypothesis of zero mean tested by t-test with a robust standard error.

Agreement of emergency medical status is unlikely to have enough events to be statistically compared. We will present cross-tabulations of (emergency detected by telemedicine center: yes/no) and (PACU nurse: disagree, investigate and agree, already aware, physician contacted). The absolute rate of telemedicine center false positives (team disagrees), true positives (team unaware), true positives (team aware), and false negatives (team aware > 15 minutes prior or t never detects) will be presented with 95% confidence intervals.

### Data collection

Multiple sources will be utilized for data collection from which outcome measures will be extracted. Data from AlertWatch will be automatically logged to a secure database.

Preoperative patient characteristics, comorbidities, surgical and clinical history, as well as perianesthesia information will be captured using Epic Systems software (Verona, WI, USA). Prospective data will be collected from Epic Systems for the datapoints mentioned throughout the proof-of-concept study.

Relevant PACU information outlined in Table 2– Table 5 for patients in this study will be collected and entered into a REDCap database managed by Washington University. Data will not be shared with others outside the research team.

### Methodological strengths and limitations

A strength of this study is its pragmatic approach as a real-world study with measurable aims. Feasibility will be determined, and information will be provided regarding logistical implications of establishing a telemedicine solution for the PACU. Many telemedicine solutions have been implemented without considering barriers and facilitators, such as cultural and political obstacles. This study proactively addresses these concerns, which might facilitate future successful implementation and generalization of similar telemedicine initiatives. Specific functions of the PACU have been detailed, and the methods of this study will allow assessment of the ability of the telemedicine center to facilitate the accomplishment of these functions.

This study also has important limitations. First, as a proof-of-concept, it will only include two PACU bays. Thus, its applicability to a large PACU will not be resolved. Second, PACU clinicians will be aware of the initiative, which could modify their behavior during the conduct of the study. Third, as the study design is observational with a before and after approach, improvements (for example in time to discharge) cannot be causally attributed to the intervention; there could be confounding explanations. Fourth, the current discharge criteria for the PACU do not have a firm evidential foundation (there is no gold standard measure for discharge readiness), and clinician gestalt plays an important role. This limitation can be addressed through development of rigorous, reliable and practical criteria. Finally, as a single center study, results will not necessarily be broadly generalizable.

### Adverse events and safety monitoring

We do not anticipate the occurrence of significant adverse events during this study. However, the primary investigator and the study team will review any adverse events identified by the departmental quality improvement program as potentially attributable to this proof-of-concept study. The occurrence of any significant adverse events will be reported to the HRPO, and the study team and HRPO would decide together whether to halt the trial. No formal data-monitoring committee will be used. There will be no audit of trial conduct during the investigation. No interim data analysis is planned for this proof-of-concept trial unless unanticipated safety issues are identified. There are no provisions for post-trial care or compensation to patients enrolled as part of this trial, as the intervention in this proof-of-concept trial involves only the addition of real-time decision-support tools and does not change existing care models.

### Dissemination

Dissemination of the findings of this study will occur via presentations at academic conferences, journal publications, and educational materials. Data from this study will not be shared with others outside the research team, as this study is a proof-of-concept designed to evaluate the potential utility and acceptability of a telemedicine solution for the post-anesthesia care unit and will only address surrogate outcomes.

### Study status

This study transitioned from the observation phase to the interaction phase in September 2020.

## Conclusions

Recovery in the PACU is an important phase in most patients’ surgical course. In this study, we propose a new model for future PACU care. Thought has been given to assess important barriers to and facilitators for the implementation of a telemedicine solution for the PACU. Potential key findings of this study might include decreased length of stay for patients in the PACU, as well as acceptance by identified key stakeholders of the telemedicine solution. Following successful pilot implementation of a telemedicine solution for the PACU, we subsequently intend to expand this model to more PACU bays, and possibly other PACU locations in order to study relevant clinical outcome measures.

The impact of this this study, and subsequent future studies, may be far reaching. The current PACU model is not well defined. A telemedicine solution for this important recovery environment has the potential to improve safety, clinical outcomes, and quality of care for patients recovering from invasive procedures. A telemedicine solution for the PACU might also provide a suitable solution for PACU environments in under-resourced or remote locations, and decrease healthcare costs for hospital systems.

## Data availability

### Underlying data

No underlying data are associated with this article.

### Extended data

Figshare: Supplemental Material for Proof-of-Concept PACU Telemedicine Protocol. https://doi.org/10.6084/m9.figshare.12944489.v1 ^16^

This project contains the following extended data in the file ‘PACU_Telemedicine_Supplement.docx’:

- PACU Telemedicine Patient Care Survey – Nurse Version
- PACU Telemedicine Patient Care Survey – Telemedicine Center Version
- PACU Telemedicine OR to PACU Hand-off Survey
- PACU Hand-off Checklist for Telemedicine Center Use
- Handoff Communication Assessment Tool of Weinger and Others

Data are available under the terms of the Creative Commons Attribution 4.0 International license (CC-BY 4.0).
