# Supplementary material for: Protocol for a proof-of-concept observational study evaluating the potential utility and acceptability of a telemedicine solution for the post-anesthesia care unit
Source: F1000Res. 2020 Oct 20;9:1261. [Version 1] doi: 10.12688/f1000research.26794.1 (PMC7656276; doi:10.12688/f1000research.26794.1)
Supplement: Peer review history

# Peer review: Reviewer response for version 1

**DOI:** 10.5256/f1000research.29587.r73442

**Published:** 2020-11-10

**Authors:** Michael Burns

**Affiliations:** 1 Department of Anesthesiology, University of Michigan, Ann Arbor, MI, USA

This is a well-designed protocol for a proof-of-concept observational telemedicine PACU study. The protocol is broken into two phases: observation and interactive, in which clinicians in the PACU and those in a telemedicine unit will determine and document important patient events. In the observation phase, the telemedicine clinicians will not interact with PACU clinicians unless there is a patient safety event. In the subsequent interactive phase, the telemedicine and PACU teams will interact. Important outcomes include determining readiness for PACU discharge.  This protocol will test the utility of telemedicine teams, use of audio-visual communication technologies, and functional implementation of decision-support software. 

 The following potential limitations are for general consideration and this study is scientifically sound without addressing these potential limitations.

1. The study size of 500 patients may limit the number of tested outcomes in the study. Rare events may be difficult to capture.
2. Assessment surveys may be difficult to accurately identify stakeholder attitudes and may be limiting in determining actionable workflow adjustments.

Overall this is an excellent study protocol that should provide high-yield results in medical support technology implementation and specific integration of clinical-support technologies to improve PACU functions. Demonstrating the potential utility of a telemedicine center to assist in PACU functions is important (Aim 1), and identifying barriers to implementation (Aim 2) may prove to be the invaluable to those attempting to implement similar technologies in medical practice.

Is the study design appropriate for the research question?

Yes

Is the rationale for, and objectives of, the study clearly described?

Yes

Are sufficient details of the methods provided to allow replication by others?

Yes

Are the datasets clearly presented in a useable and accessible format?

Not applicable

Reviewer Expertise:

Computer science and medical technology implementation, database research, clinical informatics and analytics, machine learning, natural language processing

I confirm that I have read this submission and believe that I have an appropriate level of expertise to confirm that it is of an acceptable scientific standard.

# Peer review: Reviewer response for version 1

**DOI:** 10.5256/f1000research.29587.r73445

**Published:** 2020-10-23

**Authors:** Alexandre Joosten

**Affiliations:** 1 Department of Anesthesiology, Erasme Hospital, Brussels, Belgium

This is an excellent and innovative proof of concept study ( before -after design) on the application of telemedicine in the PACU.

 The protocol is clear, well written, original, innovative and will be conduct by renowned experts.

 Excellent protocol.

 Abstract and intro are clear

 Methods are appropriate to conduct the study

 All tables and figures are very explanatory and illustrative

 No further comment. 

 Congratulations.

Is the study design appropriate for the research question?

Yes

Is the rationale for, and objectives of, the study clearly described?

Yes

Are sufficient details of the methods provided to allow replication by others?

Yes

Are the datasets clearly presented in a useable and accessible format?

Yes

Reviewer Expertise:

Technology, automated systems and artificial intelligence in anesthesia and critical care

I confirm that I have read this submission and believe that I have an appropriate level of expertise to confirm that it is of an acceptable scientific standard.
